# Supplementary material for: Prediction of polyproline II secondary structure propensity in proteins
Source: R Soc Open Sci. 2020 Jan 15;7(1):191239. doi: 10.1098/rsos.191239 (PMC7029904; doi:10.1098/rsos.191239)
Supplement: Supplementary Data and Figures [file rsos191239supp1.pdf]

ROYAL SOCIETY OPEN SCIENCE submitted

Supplementary Data
Prediction of polyproline II secondary structure propensity

Kevin T O'Brien, Catherine Mooney, Cyril Lopez, Gianluca Pollastri and Denis C. Shields

Evaluating Performance

To evaluate the performance of PPIIPred we measured the true positive rate (TPR) and false positive rate (FPR) as we increased the discrimination threshold from 0 to 1. The results are shown as a Receiver Operating Characteristic (ROC) curve where TPR is plotted against FPR, which were calculated as follows:

$$
\begin{aligned}
TPR &= \frac{TP}{TP+FN} \\
FPR &= \frac{FP}{FP+TN}
\end{aligned}
$$

where:

- True positives (TP): the number of residues predicted in a class that are observed in that class.
- False positives (FP): the number of residues predicted in a class that are not observed in that class.
- True negatives (TN): the number of residues predicted not to be in a class that are not observed in that class.
- False negatives (FN): the number of residues predicted not to be in a class that are observed in that class.

The area under the curve, AUC, which is equivalent to the probability that the classifier will rank a randomly chosen positive instance higher than a randomly chosen negative instance (Fawcett, 2006) is also shown. The AUC is a number between 0 and 1 where 0.5 indicates a random model and 1 is perfect. Python code was used to plot the curves and calculate the AUC. Specificity (Spec), sensitivity (Sens), Matthews Correlation Coefficient (MCC) and the accuracy (Acc) at a 0.2 threshold are measured as follows (Baldi *et al.*, 2000):

$$
\begin{aligned}
Spec &= 100\frac{TP}{TP+FP} \\
Sens &= 100\frac{TP}{TP+FN} \\
MCC &= \frac{TP\times TN - FP\times FN}{\sqrt{(TP+FP)(TP+FN)(TN+FP)(TN+FN)}} \\
Acc &= 100\frac{TP+TN}{TP+TN+FP+FN}
\end{aligned}
$$

MCC is an indicator of overall performance between the observed and predicted classifications for both PPIIH and other residues. A value of 1 represents a perfect prediction, 0 a random prediction and -1 a completely inverse prediction.

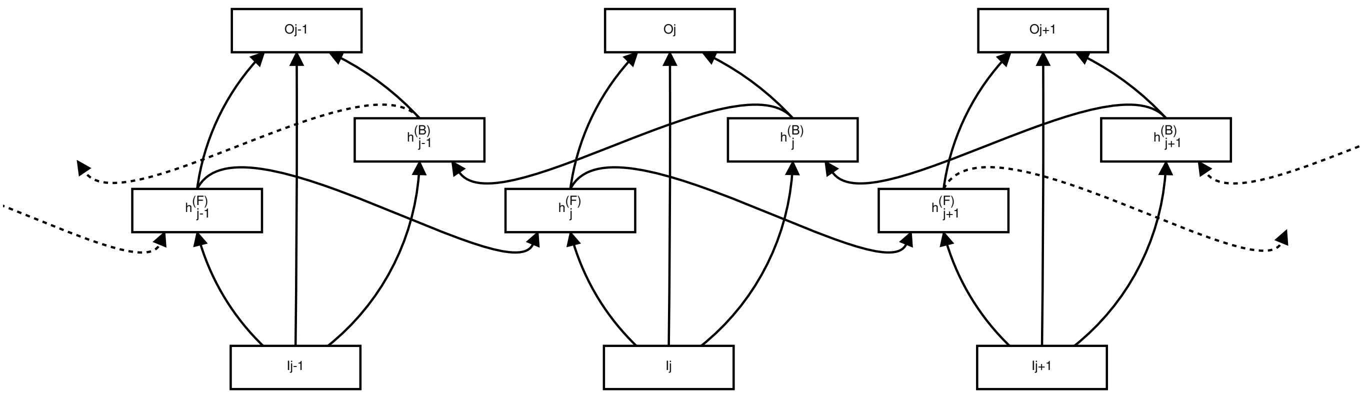

Figure S1: **Graphical representation of a BRNN.** Rectangles represent input, hidden and output vectors. Arrows represent functional dependencies, for example o_j is a function of i_j, $h_j^{(F)}$ and $h_j^{(B)}$; $h_j^{(F)}$ is a function of i_j and $h_{j-1}^{(F)}$; etc. Terminal states $h_0^{(F)}$ and $h_{N+1}^{(F)}$ (not represented) complete the graphical model. Notice that any input can, in principle, affect any output.

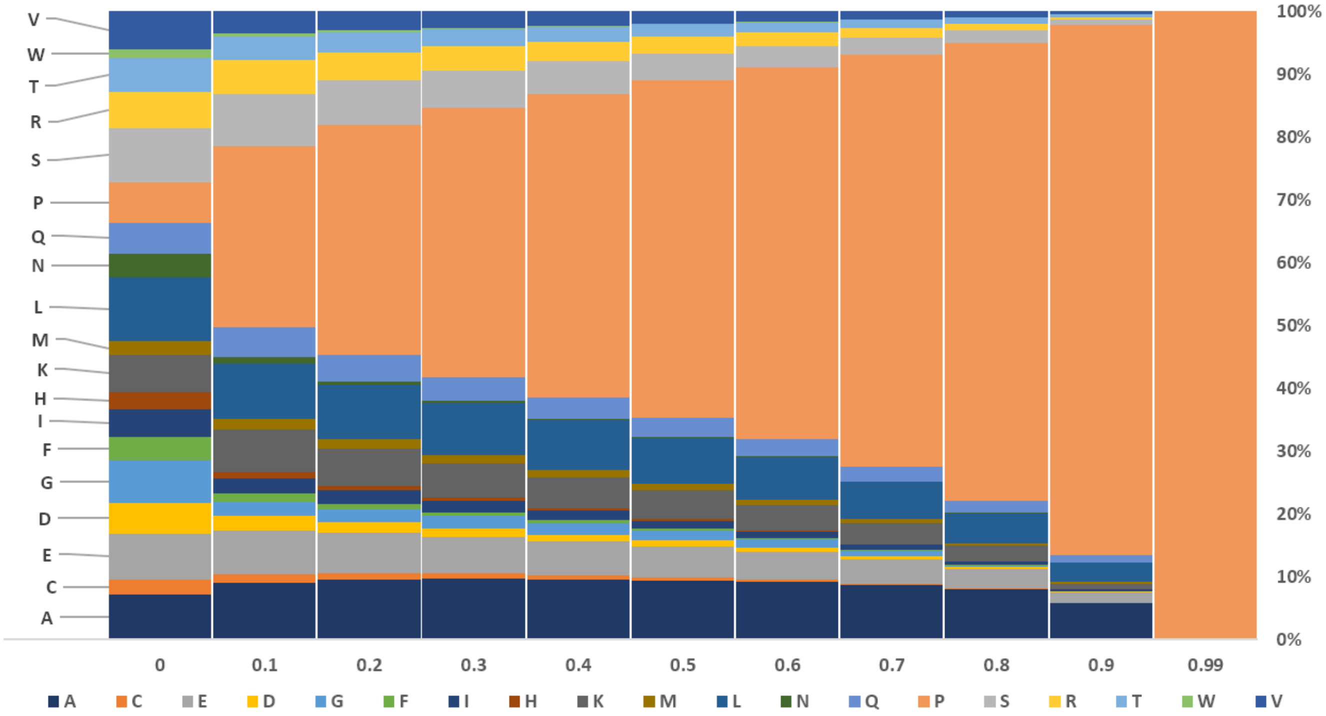

Figure S2: **Amino Acid Composition** (Left) The PPIIH amino acid composition of the TP (strict) dataset. (Right) Predicted amino acid composition of PPII predictions in the human proteome at various cutoffs.

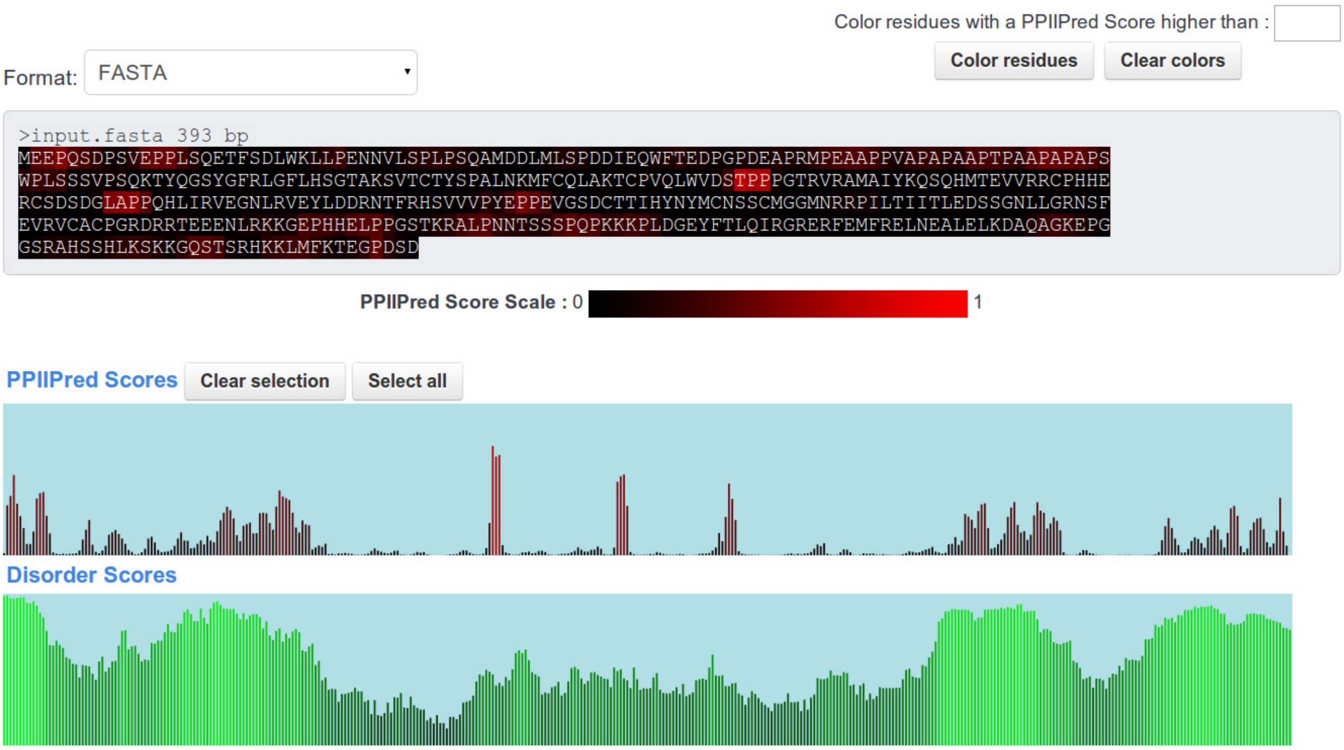

Figure S3: **The PPIIPred results interface.**

References

Baldi, P. *et al.* (2000). Assessing the accuracy of prediction algorithms for classification: an overview. *Bioinformatics*, **16**(5), 412–424.

Fawcett, T. (2006). An introduction to ROC analysis. *Pattern Recogn Lett*, **27**(8), 861–874.
